# A less visible pandemic

**DOI:** 10.1016/j.lanwpc.2020.100035

**Published:** 2020-09-24

Since its emergence in December, 2019, COVID-19 has substantially changed the world we live in: people are dying, daily life is radically affected, and society has gone into a state of hibernation. At a time when COVID-19 attracts all our attention, another pandemic continues to destroy the lives and livelihoods of people, quietly.

Like infectious diseases, non-communicable diseases (NCDs), mainly cardiovascular diseases, chronic respiratory disease, cancer, and diabetes, kill 41 million people each year. In the Western Pacific region, NCDs account for 80% of all deaths, with 50% of premature deaths occurring in low-income and middle-income countries. Most NCDs have preventable risk factors such as an unhealthy diet, low amounts of physical activity, tobacco use, and alcohol consumption. Those behaviours are influenced by the socioeconomic status of each individual, and the effect is most visible in the most susceptible groups. This phenomenon is very concerning in Pacific island countries, where the prevalence of obesity and diabetes is the highest in the world. Additionally, NCD health service delivery is hampered by geographical isolation and a shortage of financial support and human resources. In The Lancet’s NCD Countdown 2030, published on Sept 3, 2020, the likelihood of dying from NCDs is less than 6% in women from high-income countries such as Australia, Japan, Singapore, and South Korea, whereas Papua New Guinea is among the countries with the highest NCD mortality. Even though the overall trend is decreasing in most countries, only three high-income countriesNew Zealand, Singapore, and South Koreaare on track for achieving the 2030 objective of reducing premature deaths by a third from NCDs.

People with NCDs are at a higher risk of becoming severely ill or dying with COVID-19. But the effect of COVID-19 on NCDs is more than clinical. As most NCDs are chronic, continuous health-care services and medical supplies are often required. In a survey of 163 member countries in May, 2020, WHO reported that health-care services for NCDs were disrupted in most countries during the COVID-19 pandemic. The management of hypertension was affected in 53% of countries and the management of diabetes was affected in 49% of countries. The situation is slightly better for cancer treatment and cardiovascular emergencies, but still around 42% of countries had disruption to cancer treatment and 31% of countries showed disruption during cardiovascular emergencies. According to the report, staff shortages, medical supply shortages, and closed health facilities are the main reasons for discontinuation of NCD-related health-care services. Along with health-care service disruption, COVID-19 mitigation strategies in many countries, such as lockdown, physical distancing, and self-isolation, increase the behavioural risk for NCDs. The interruption of regular health services and medical supplies, coupled with unhealthy lifestyle changes, pose a new challenge for people living with NCDs.

As we are preparing to live with COVID-19 for the foreseeable future, all stakeholders need to make efforts to protect and support people with NCDs. To include NCDs in national COVID-19 responses, to ensure the continuous health care and medical supply for disease management, and to prevent risk factors such as reducing tobacco and alcohol use are some immediate strategies to protect people with NCDs from the current crisis. Going forward, a more accessible and resilient health-care system is needed to reduce the proportion of susceptible populations and to be better prepared for future pandemics.

Health information and data are key in this process. In The Lancet Regional Health Western Pacific, Matthew Hare and colleagues present a report on the increased prevalence of maternal hyperglycaemia in a cohort of Aboriginal and non-Aboriginal women in the Northern Territory of Australia from 1987 to 2016. The study provides crucial data for policy makers and Indigenous communities to develop effective approaches to reduce metabolic diseases during pregnancy and improve the health and wellbeing of mothers and newborn babies. In another pluri-ethnic survey study in New Caledonian adolescents, Stephane Frayon and colleagues found that Melanesian and Polynesian adolescents are at a higher risk of being overweight compared with European adolescents. Ethnic background and socioeconomics are the major contributors to these NCDs. This area is part of the journal’s mission to be the platform for the publication of regional research on NCDs, with the hope of accelerating the treatment and management of this long-lasting silent pandemic.

*The Lancet Regional Health – Western Pacific*

